# Raptor: A Design of a Drain Inspection Robot

**DOI:** 10.3390/s21175742

**Published:** 2021-08-26

**Authors:** M. A. Viraj J. Muthugala, Povendhan Palanisamy, S. M. Bhagya P. Samarakoon, Saurav Ghante Anantha Padmanabha, Mohan Rajesh Elara, Dylan Ng Terntzer

**Affiliations:** 1 Engineering Product Development Pillar, Singapore University of Technology and Design, 8 Somapah Rd, Singapore 487372, Singapore; viraj_jagathpriya@sutd.edu.sg (M.A.V.J.M.); povendhan_palanisamy@mymail.sutd.edu.sg (P.P.); saurav@sutd.edu.sg (S.G.A.P.); rajeshelara@sutd.edu.sg (M.R.E.); 2 LionsBot International Pte. Ltd., #03-02, 11 Changi South Street 3, Singapore 486122, Singapore; dylan@lionsbot.com

**Keywords:** drain inspection, inspection robotics, reconfigurable robotics, navigation control, public health and safety

## Abstract

Frequent inspections are essential for drains to maintain proper function to ensure public health and safety. Robots have been developed to aid the drain inspection process. However, existing robots designed for drain inspection require improvements in their design and autonomy. This paper proposes a novel design of a drain inspection robot named Raptor. The robot has been designed with a manually reconfigurable wheel axle mechanism, which allows the change of ground clearance height. Design aspects of the robot, such as mechanical design, control architecture and autonomy functions, are comprehensively described in the paper, and insights are included. Maintaining the robot’s position in the middle of a drain when moving along the drain is essential for the inspection process. Thus, a fuzzy logic controller has been introduced to the robot to cater to this demand. Experiments have been conducted by deploying a prototype of the design to drain environments considering a set of diverse test scenarios. Experiment results show that the proposed controller effectively maintains the robot in the middle of a drain while moving along the drain. Therefore, the proposed robot design and the controller would be helpful in improving the productivity of robot-aided inspection of drains.

## 1. Introduction

Drains are a crucial infrastructure in every contemporary city to mitigate flooding at the surface and to remove excess water or sullage in an obscured and efficient way. Stormwater drains mitigate water flow while sewage mitigates sullage flow. Sometimes, drains are combined to transfer stormwater and sullage [1,2]. Flooding can damage private property and public water supply infrastructure causing contamination in water reservoirs [1,2]. Drainage networks in most countries are mostly underground. The benefits of drainage systems are the continuous water supply, distribution, and storage with minimal wastage, and controlled sullage disposal. However, drainage systems also come with many challenges [3]. Drains require regular inspections to monitor slit level, structural maintenance due to exposure or damage and routine dredging for free-flowing drains [4]. It is common to find clogging, even in new drains, that can result in water stagnation and create mosquito breeding sites and thus they need to be cleaned frequently to reduce the risk of malaria and dengue [5]. Coupled with pathogenic pollution in drainage systems, the human labour needed to maintain them may be deemed unsafe and costly [6].

Frequent inspections should be conducted to mitigate the issues mentioned above. Several robotics solutions were introduced to resolve issues associated with the maintenance and inspection of built environments [7,8,9,10]. Similarly, robots have been used for the inspection of confined spaces such as pipes and air ducts [11,12]. In [13], the authors introduced several types of inspection robots that are used for pipe inspections, such as long-distance inspection for sewer pipes. Apart from these robotics solutions, robot aided inspection methods play a vital role in urban infrastructure inspection and maintenance [14].

Drain inspection robots are specifically designed for internal drainage system geometry and can be classified into three forms, namely: tracked, wheeled, and legged [15]. Over time, drain inspection robots have been evolved into autonomous and semi-autonomous robots to reduce human involvement in inspection and cleaning. For example, PIRAT [16] is a semi-autonomous pipe vehicle that surveys sewer systems quantitatively in real-time using artificial intelligence to measure the drain geometry and detect defects. KARO [17] is another semi-autonomous wheeled tethered robot with sophisticated multi-sensors for smart sensor-based sewage inspection. KANTARO [18] is an untethered autonomous wheeled platform that can access straight and even bendy pipes without the use of sensors or controllers using a specific mechanism known as the “naSIR Mechanism”. KURT [19] is an untethered autonomous six-wheeled vehicle that can fit into 600 mm diameter pipelines. MAKRO [20] is an untethered autonomous worm-shaped wheel, multiple segments, and an autonomous engine for the navigation of drainage systems. However, these robots are unable to adapt to varying drain dimensions and morphology. Tarantula [15,21] is a self-configurable drain mapping robot that can adapt and morph according to a drain that has multiple level shifts. This robot has a complex mechanical design with many actuators that lead to a high cost of implementation and are difficult to control. In addition, the robot’s dimensions are large where the manoeuvrability within some drain segments is difficult.

The condition assessment or the classification of the fault can be generally divided into sewer and water pipelines [22]. Sewer pipeline faults are subdivided into blockages, cracks, sag, corrosion, collapsed mortar, open-joints, and root intrusion, while water pipeline faults are subdivided into deformations, leakage, dislocation, corrosion and fractures. This hierarchy can serve as a general guideline when developing algorithms to identify faults in drainage systems. The development of deep learning techniques in computer vision have proven to be effective in these condition assessments. In this regard, Deep Convolutional Neural Networks (CNNs) have been explored to recognize cracks and defects [23]. In the cited work, a CNN is used to construct high-level features from low-level features that are then utilized by a multi-layer perceptron to perform detection. An alternative approach was to use only CNNs to recognize cracks in the absence of the conjugation of image processing techniques. However, this model needed a large dataset and was computationally straining [24]. To keep costs down, vision cameras are often used as opposed to expensive Lidar or ultrasound sensors [25]. Stereoscopical and monocular camera-based vision systems are also used for object detection. A stereoscopical algorithm in the field of autonomous vehicle navigation was created by Broggi et al. [26] using V-disparity image computation, pitch estimation, disparity image computation, obstacles localization, and real-world coordinates mapping. This algorithm was robust in computing the camera pitch angle during data acquisition. However, it needed a further development of individual color channels to reduce false positives. Notably, a monocular camera-based algorithm for Unmanned Aerial Vehicles (UAVs) was suggested by Al-Kaff et al. [27] to detect object size change, and the ratio of the size expansion is computed using UAV movement, which performed well only under uniform lighting conditions. The limitations of these algorithms seem to be the lack of general usability in lighting variations. However, the scope of the work cited above is limited to the development of algorithms for computer vision aided condition classification and detection, and the design and development of robots and control features are not discussed.

This paper proposes a novel design of a drain inspection robot, Raptor, equipped with the autonomous drain following functionality. Reconfigurability of a robot can eliminate some of its limitations and increases the robot’s capabilities [28,29]. Thus, the mechanical system of the robot is designed to facilitate manual reconfigurability that can be used to adjust the ground clearance per the requirements. The robot has been designed in such a way that it satisfies all the essential features required for a drain inspection robot where the existing robots have limitations. This design consideration is the core contribution of the proposed design with respect to the state-of-the-art. The autonomous drain functionality has been developed using a fuzzy controller, which makes control actions maintain the robot in the middle of a drain while moving along the drain. The particulars of the mechanical, control, and autonomy design aspects of the robot are presented in Section 2. The fuzzy controller proposed to navigate the robot in the middle of a drain is explained in Section 3. Experimental validation is discussed in Section 4. The conclusions of the work are given in Section 5.

## 2. Robot Design

In this section, the details of a robot platform designed for drain inspection purposes are discussed. The design principles, reconfigurability, structural analysis, control, and autonomy layers of the robot are also briefed.

### 2.1. Mechanical Design

#### 2.1.1. Design Principle

The design requirements considered for the drain inspection robot, Raptor, are derived based on the literature study of existing drain inspection robots, their limitations, and design considerations. The requirements are as follows: (a) ability to manoeuvre in narrow drains; (b) suitable to travel in various terrain; (c) lightweight and compact; (d) capability to make Sharp turns; (e) flexibility to mount sensors and actuators; and (f) capability to carry a payload. All these features were considered while designing the platform to be versatile and robust for drain inspection applications. In contrast, the existing robot designs are not in compliance with all these requirements. Comparisons between the existing robots and the proposed design based on these design considerations are given in Table 1 to highlight the improvements of the proposed design.

**Figure 1:**
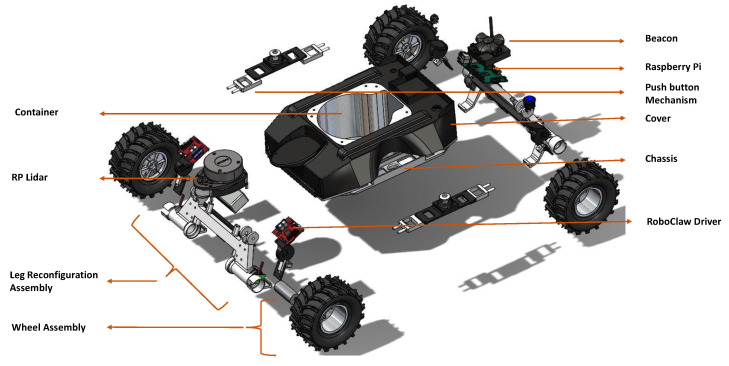
Mechanical components.

Figure 1 shows an exploded view of Raptor, and the primary components are annotated. The backbone structure of Raptor, the chassis, is 3D printed in Nylon for greater flexibility, durability, and higher tensile strength. The chassis is designed with a cylindrical carriage for payload at the centre. All other components are designed around it to stabilize the platform by placing the centre of gravity in the middle. The structure holding the wheels to the chassis is manufactured with additional thickness to bear the direct impulse forces from the ground. All the wheels are individually powered by geared motors. The 12 V DC motors with carbon brushes and a 391:1 gear ratio deliver 5 kg-cm torque. With a diameter of 20 mm, the motor is compact and power-packed. All-wheel drive enables faster turning and easier obstacle crossing.

Twenty cm rubber wheels with thicker grips offer the friction stick-slip grip required for the wet drain terrain. Magnetic wheel encoders are fixed with the drive motors to take velocity and position feedback. With a 390 × 350 × 200 mm platform dimension and form factor, the Raptor can cope with a maximum vertical gradient of 20–25${}^{\circ }$. The push-button mechanism and the pull lever mechanism work in combination to reconfigure the position of the wheels. The Lidar module acts as the primary sensor for perception and control.

#### 2.1.2. Reconfiguration Module

**Figure 2:**
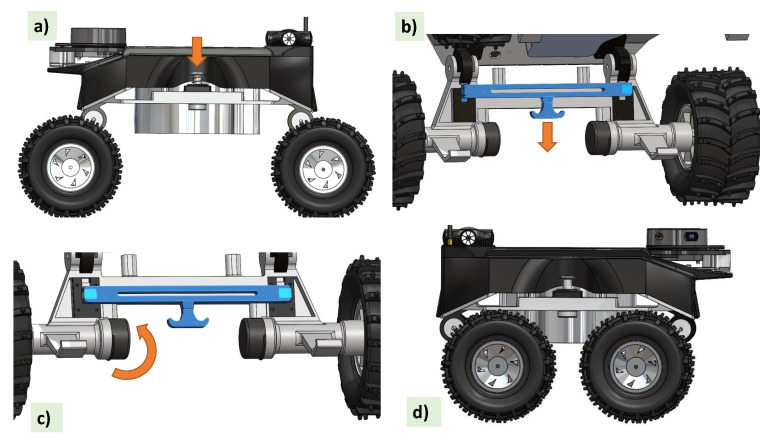
Steps of the reconfiguration. (**a**) Push button pressing to open the provision to insert and hold the pin (**b**) Pulling the lock open when triggering the pull lever (**c**) Adjusting the wheel assemblies (**d**) New wheel assembly configuration

As depicted in Figure 2, the reconfiguration for the Tuck-In and Tuck-out mechanisms is triggered through a combination of the push-button mechanism and the pull lever mechanism. Step (a), press the button when the spring is pushed down to open the provision to insert and hold the pin in place; in the next step (b), when the pull lever mechanism is triggered, it pulls the lock open on both wheels; (c) now the wheel assemblies are free to be rotated around the reconfiguration axis as shown in Figure 2; finally, when the pin is in place, both the mechanisms are released, and the wheels are secured with a modified height and length with respect to the Raptor. The ground clearance for the Tuck-In mechanism is 7 cm and for the Tuck-out mechanism it is 11 cm. With this variable ground clearance option, the configuration is used appropriately depending on the situational demands.

**Figure 3:**
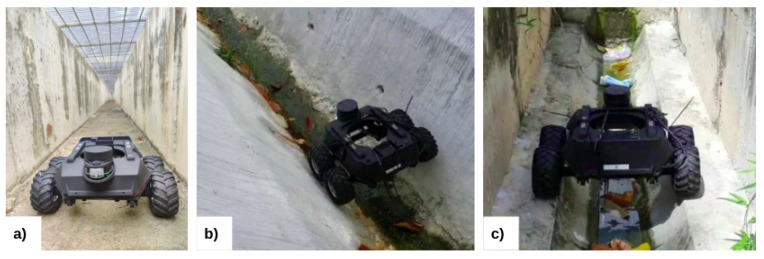
Adaptability of the robot to various drain scenarios. (**a**) In a drain with a flat floor (**b**) In a drain with a curved bottom (**c**) In a cut-off drain

The reconfiguration ability allows the robot to adapt to different drains. Various deployments, including drains with flat floors (as shown in Figure 3a) which are designed for excessive flood water drainage, open drains with a curved bottom as shown in Figure 3b, and cut-off drains as shown in Figure 3c have been considered to demonstrate the adaptability of Raptor. The demonstrations show that flat drains with concrete floors need more traction, control and speed. For this application, Raptor with tucked-in configurations is identified as more suitable because of its rigid dynamics and the structural stability of the configuration. For the cases of drains with wide separation and water flow in the middle, the tuck-out configuration is identified as being more suitable with its structural flexibility.

#### 2.1.3. Kinematics

**Figure 4:**
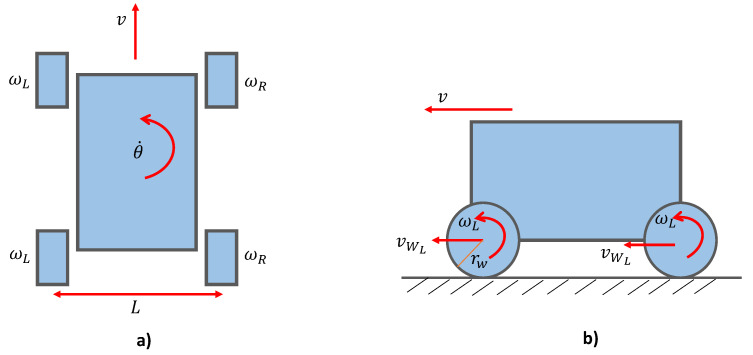
Kinematics. (**a**) Top view (**b**) Side view

This section describes how high-level velocity commands are converted to low-level wheel angular velocities through the kinematic model. The free body diagram of Raptor representing the linear velocity, *v*, and angular velocity, $\dot{\theta }$, of the robot are shown in Figure 4. *L* represents the distance between the left and the right wheels, and $V_{W_{L}}$ and $V_{W_{L}}$ represent the linear velocities of the left and right wheels, respectively.

Since the wheels on the side are driven in parallel, the angular velocity of Raptor is varied through the difference in angular velocity for the right side $\omega_{R}$ and left side wheels $\omega_{L}$. The two wheels on the inside spin with a lower velocity compared to the outer wheels when making a turn. The difference in velocity determines the radius of curvature of the turn. For an in-pivot turn, that is, rotating around its centre, both wheels spin in the opposite direction with the same magnitude. Angular velocities of individual wheels for a given velocity command *v* and $\dot{\theta }$ are calculated through the simple kinematic model given in (1), where $r_{W}$ is the wheel radius. These velocity commands are provided by the fuzzy logic controller explained in Section 3.

(1) $$\begin{array}{c} \omega_{R}=\frac{v}{r_{W}}+\frac{\dot{\theta }}{2r_{w}}L\\ \omega_{L}=\frac{v}{r_{w}}-\frac{\dot{\theta }}{2r_{w}}L. \end{array}$$

#### 2.1.4. Structural Analysis

**Figure 5:**
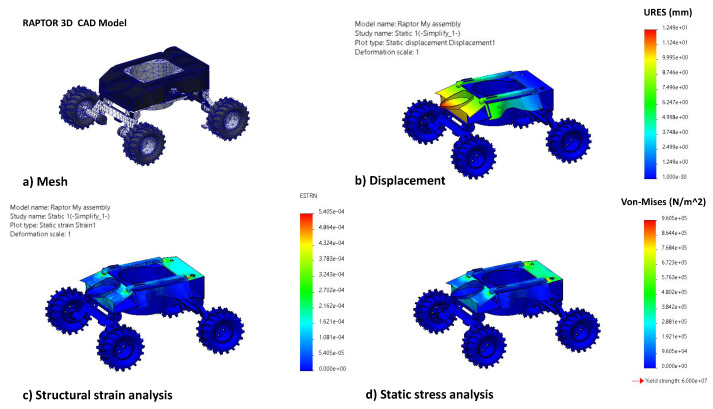
Static structural analysis (**a**) Tetrahedral mesh of Raptor 3D model (**b**) Static Displacement analysis (**c**) Strain Analysis results (**d**) Von Mises stress in N/m${}^{2}$.

The structural load-bearing capacity of the platform is an essential factor for a rigid and robust platform design in-order for robust operation in the drain environment. It is identified that the critical scenario for failure is the head on collision of Raptor with the drain walls. This event could impose severe stress on the structure and could potentially break the weakest link. Hence, this scenario is simulated with Finite Element Analysis and cross-checked for maximum stress and strain of the structure. For this simulation, the CAD model was meshed with a tetrahedral mesh as shown in Figure 5. Boundary conditions were applied with fixed constraints on all the four wheels and a static load of 10N was applied on the front impact region of the vehicle. A static structural analysis was performed in order to analyse the structure based on displacements (refer to Figure 5b), maximum von-mises (refer to Figure 5c) stress and enduring strain (refer to Figure 5d).

The tensile strength of Nylon material is 60 MPa, and the results show that the max stress concentration is safely lower than the maximum allowable stress.

### 2.2. Control Architecture

#### 2.2.1. Electronics and Control

**Figure 6:**
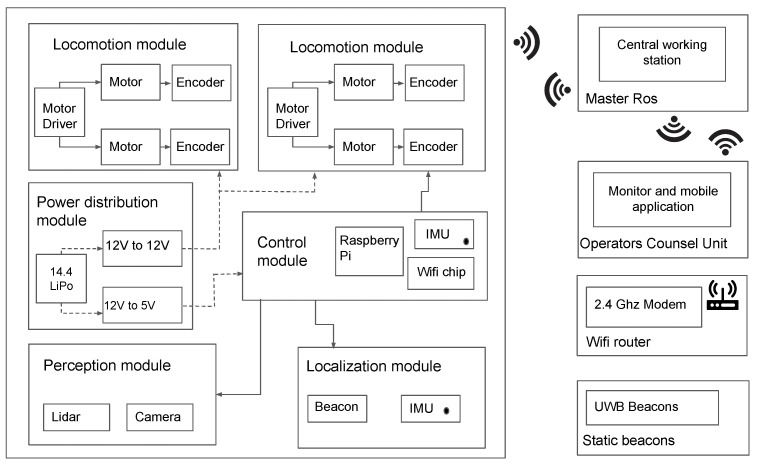
Control Architecture.

The electronics and control architecture of the robot are depicted in Figure 6. The locomotion module consists of four DC metal-gear motors (with 391:1 gear ratio and the dimension of 20D × 46L mm). Motor pairs on each side mounted to the chassis structure are controlled by separate motor drivers (RoboClaw) with separate addresses through the Universal Serial Bus (USB) protocol. This communication is to facilitate the four-wheel independent drive motor control, and the USB protocol allows us to publish data to motors and subscribe the feedback from encoders. The leg provision in the chassis provides a secure mounting for magnetic wheel encoders. The perception for Raptor is primarily through 2D Lidar (RPLidar A1M8) with the USB protocol. Lidar facilitates the mapping of the environmental situation and navigation in drains. A 4-cell Lithium-ion battery (14.4 V, 2800 mAh) is used to power the 5 V Single board computer (SBC) and 12 V motor drivers through respective voltage regulators. The remaining sensors are either powered through USB or Input/Output (I/O) circuit connections through the SBC. The SBC was chosen carefully based on multiple considerations. A RaspberryPi microprocessor with a clock speed of 1.2 GHz outperforms the Arduino microcontroller, which has a clock speed of 16 MHz. Furthermore, our application demands a microprocessor with inbuilt CPU processor, RAM, Graphics card, and connector pins. In addition to performing the high level ROS autonomy processing, RaspberryPi also enables direct communication with motor drivers and IMU through the USB and I/O communication protocols. Even though more powerful microprocessors like Jetson Nano, with its high graphical processing capabilities, easily overpowers RaspberryPi, a more optimised SBC solution must be chosen considering power consumption, cost and processing requirements. Since the data being processed is of moderate heaviness, RaspberryPi Model B is chosen as the appropriate SBC. A 435i RealSense camera unit is mounted to RaspberryPi, and the data are directly streamed to the ROS master unit on a high processing Personal computer (PC) with a Graphics Processing Unit (GPU) for the machine learning layer. RaspberryPi firmware is installed with Ubuntu 18.4. All the sensors, SBC, and the GUI control module are integrated through the ROS (Robot Operating System) framework. ROS operates with all the sensors (slave node) and SBC (master node) acting as nodes communicating through ROS topics with customized message formats.

#### 2.2.2. Autonomy Layer and Graphical User Interface (GUI)

**Figure 7:**
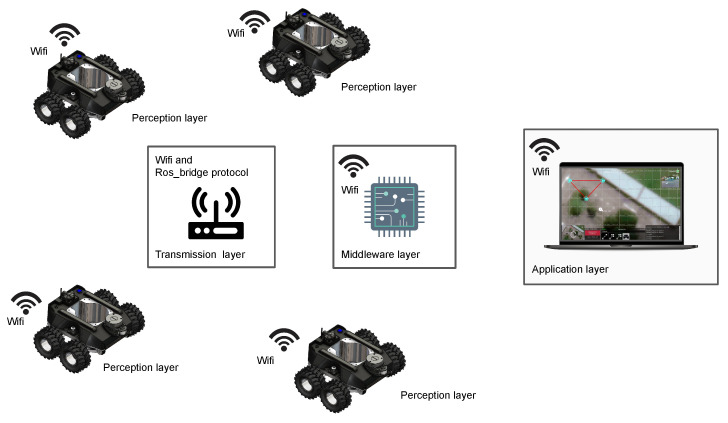
Overview for a Raptor drain inspection system.

Raptor is designed to be teleoperated by users through mobile or laptop applications connected to the same network as the ROS master. This multi-device compatible Graphical User Interface (GUI) application is developed using Unity 2019.4, a cross-platform engine for developing graphical programs. The communication between robot and control devices happens via a Message Queuing Telemetry Transport (MQTT) bridge through ROS. In the communication protocol, ROS messages are serialized by JavaScript Object Notation (JSON) for MQTT communication and deserialized back for ROS messages. As shown in Figure 7, the GUI counsel has a provision for video feed and control buttons that equip basic navigation operations based on the visual input through the camera feed from the robot. The user can also choose to navigate along a drain autonomously. In this regard, the user activates the drain following functionality through the GUI. In that case, the proposed fuzzy logic controller (explained in Section 3) is activated, and the robot can autonomously move along the drain while maintaining its position in the middle. This autonomous behaviour avoids the overhead for the operator where the users have to perform teleoperation only for supervisory control actions such as pausing, stopping, and changing the direction required for returning, careful inspections of vigilant locations, in a junction of a drain, and unforeseen events. A 2D Lidar sensor is used for perceiving the environment to ensure obstacle detection and avoidance. An additional safety layer is added to avoid unintentional obstacle collision due to the carelessness of the operator. Measurements of wheel encoders in combination with the Inertial Measurement Unit (IMU) are used for the position and heading angle estimation. This method provides a reliable localization for Raptor.

## 3. Navigating in the Middle of a Drain

The main intention of the robot is to inspect the drainage. The robot should be capable of navigating in the middle of the drainage for safety and better inspection of the surrounding. A robot operates in a drain often subjected to a lot of external disturbances due to the uneven nature of the terrain. Furthermore, there is a limited space in a drain, and the robot should maintain a proper clearance with side walls while moving. Thus, the robot has been incorporated with a fuzzy logic controller to maintain the robot in the middle of a drain during the navigation.

Fuzzy logic is considered to be an intelligent technique to cope with a system with unknown dynamics [30,31,32]. This technique can be applied for a complex system defined with linguistic expressions that has a non-linear relation with the input and output [32,33,34]. According to the literature, fuzzy logic systems can be considered universal approximators [35]. The drainage robot “Raptor” dynamic model cannot be fully modeled due to the drainage frictions and the liquids, which can be taken as environmental factors. Furthermore, the dynamics of the robot vary with the wheel assembly reconfiguration. One of the advantages of the fuzzy logic controller is that it can perform well in scenarios where it has less knowledge of the underlying dynamics of the robot or the environment [36,37,38,39].

**Figure 8:**
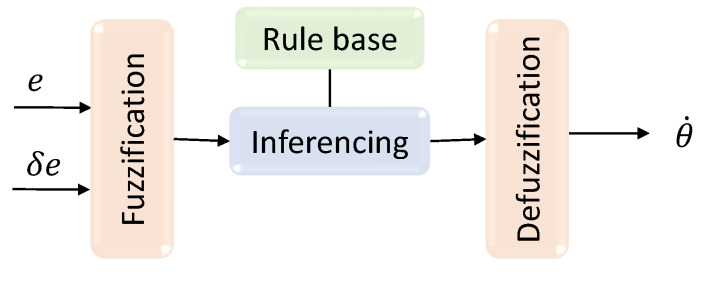
Architecture of the fuzzy logic system.

**Figure 9:**
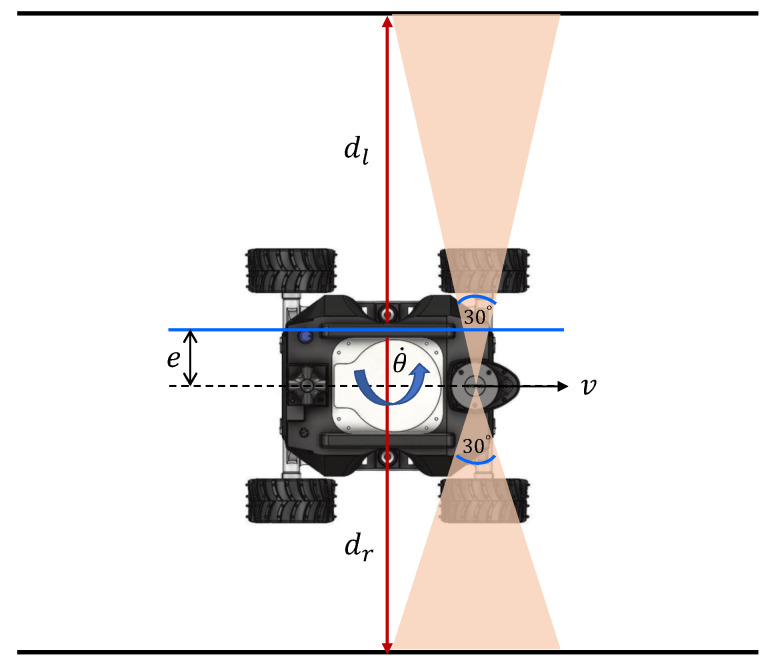
Principle of navigating in the middle of a drain.

The architecture of the proposed fuzzy controller is depicted in Figure 8. The aim of the controller is to maintain the robot in the middle of the drain for safe navigation and proper inspection coverage. The clearance of the robot with the side walls of a drain are measured based on Lidar readings. A window of 30${}^{\circ }$ in the right and left sides of the robot, as shown in Figure 9, is considered to calculate the average distance of each side, $d_{r}$ and $d_{l}$. The position of the robot inside a drain is estimated from these clearance measurements. In the event of the robot being perfectly in the middle of the drain, the clearance from the two sides are the same where $d_{r}=d_{l}$. The deviation of the robot from the middle of the drain can be calculated as in (2) for the current time step, *t*. The inputs of the fuzzy logic controller are the present deviation from the middle (defined as *e*) and the difference of the deviation during successive steps of the controlling loop, $\delta_{e}$. $\delta_{e}$ is calculated as in (3). The two parameters are chosen as the input since these parameters reflect the present and the trend of deviation of the robot from the middle of a drain. The output of the fuzzy system is the angular velocity of the robot (i.e., $\dot{\theta }$), which steers the robot to sides. The angular velocities of the right and left wheels corresponding to the reference $\dot{\theta }$ are determined from the kinematic model of the robot.

(2) $$e\left(t\right)=d_{l}\left(t\right)-d_{r}\left(t\right)$$

(3) δe(t)=e(t)−e(t−1).

**Figure 10:**
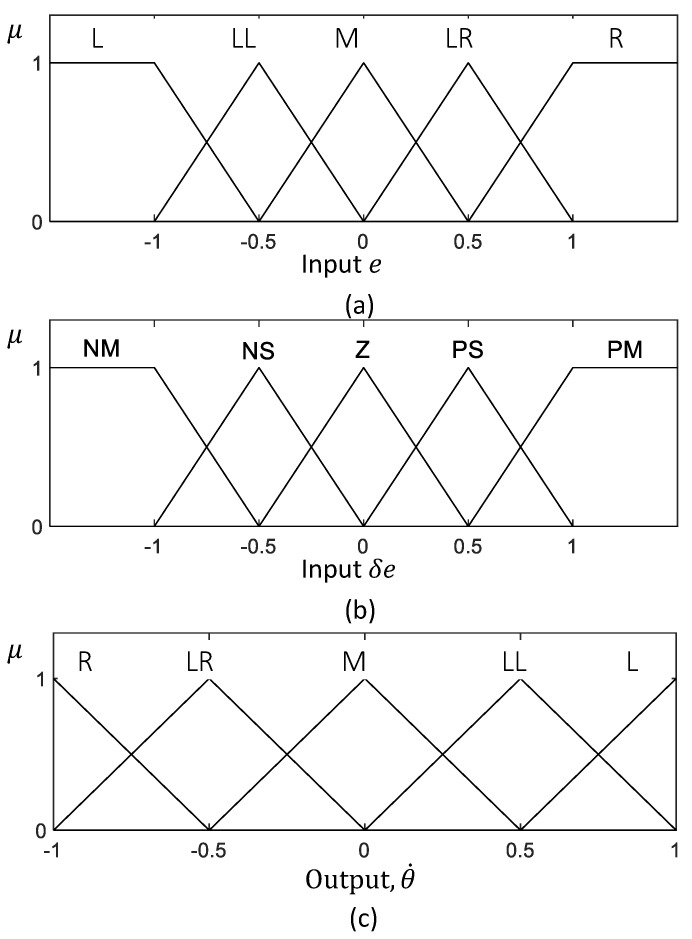
Input and output membership functions of fuzzy inference system. (**a**,**b**) The input membership functions. (**c**) The output membership function.The fuzzy labels are defined as R: Right, LR: Little Right, M: Middle, LL: Little Left, L: Left, NM: Negative Medium, NS: Negative Small, Z: Zero, PS: Positive Small, and PM: Positive Medium. It should be noted that the membership functions are given in normalized scales.

**Figure 11:**
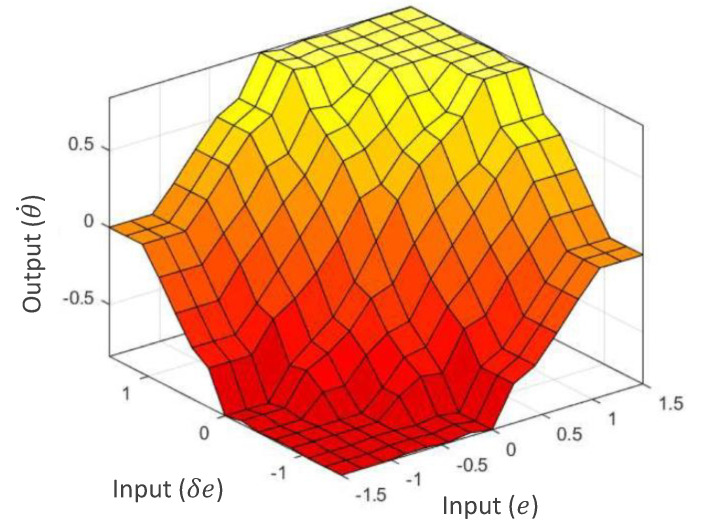
Decision surface of the fuzzy logic system

Fuzzification of the two inputs, *e* and δe, is executed according to the input membership functions depicted in Figure 10a,b. $\mu_{e}$ and $\mu_{\delta e}$ are the fuzzified inputs that correspond to *e* and δe, respectively. Fuzzy rules defined for the robot for the required control actions are given in Table 2. The rule base of the fuzzy logic system has been defined such that the robot’s actions counter the error of the robot from the middle of the drain. For example, if the robot is on the right side from the middle, the robot’s angular velocity is set to turn toward the right and vice versa. When the deviation is slight, then the amount of angular velocity for turning is also reduced. The change of error is also analyzed in the control rule to minimize the oscillations caused by overshooting. For example, if an error change indicates a quick correction, the effective robot’s corrective action is reduced to avoid overshooting. The rule base of the fuzzy inference system has been defined based on expert knowledge to reflect this example control behavior. The input fuzzy sets are mapped with the output fuzzy sets using the rule base during the interfacing. The output membership functions are depicted in Figure 10c. Minimum fuzzy t-norm and maximum t-conorm are considered in the fuzzy logic system. In the firing strength $i^{\mathrm{th}}$ rule, $f_{i}$ can be expressed as in (4). Mamdani’s minimum operation rule leads to the fuzzy consequent of the $i^{\mathrm{th}}$ rule, $\mu_{\dot{\theta }\prime_{i}}$ given in (5). The accumulated fuzzy consequents can be obtained from (6), where *n* is the number of rules. The accumulated set of output fuzzy consequents is defuzzified in the defuzzification layer. The defuzzified crisp output, ${\dot{\theta }}_{*}$, can be obtained from (7). The linear velocity is a constant. However, if the angular velocity exceeds the threshold ${\dot{\theta }}_{T}$, the linear velocity will change according to (8). These values were set that satisfy the limits of the kinematic model. The expected variation of the output of the fuzzy logic system with the inputs is shown in Figure 11.

(4) $$f_{i}=\mu_{e_{i}}\wedge \mu_{\delta e_{i}}$$

(5) $$\mu_{{\dot{\theta }}_{i}^{\prime }}=f_{i}\wedge \mu {\dot{\theta }}_{i}$$

(6) $$\mu_{{\dot{\theta }}^{\prime }}=\mu_{{\dot{\theta }}_{1}^{\prime }}\vee \mu_{{\dot{\theta }}_{2}^{\prime }}\vee ...\mu_{{\dot{\theta }}_{n}^{\prime }}$$

(7) $$\dot{\theta }*=\frac{\int_{-\infty }^{\infty }\dot{\theta }\mu_{{\dot{\theta }}^{\prime }}d\dot{\theta }}{\int_{-\infty }^{\infty }\mu_{{\dot{\theta }}^{\prime }}d\dot{\theta }}$$

(8) $$v=\left\{\begin{array}{cc} k_{v} & \mathrm{if}\;|\dot{\theta }|<{\dot{\theta }}_{T}\\ 0 & \mathrm{otherwise}. \end{array}\right.$$

## 4. Experiments and Results

### 4.1. Experimental Setup

A prototype of the proposed robot design has been developed. Experiments were conducted to validate the proposed controller designed to maintain the robot in the middle of a drain during navigation. In this regard, four test scenarios were considered by deploying the robot in a drain. Two test scenarios created in a laboratory setting were used as additional cases. The robot’s parameters were configured as follows for all the test scenarios of the experiments: The experiments were conducted by activating the autonomous drain following functionality through the GUI after placing the robot at the respective starting position. The proposed controller was used to autonomously move the robot during the test scenarios without interventions of the user other than starting and stopping. The default linear velocity of the robot, $k_{v}$, was configured to 0.08 m/s, while the threshold, ${\dot{\theta }}_{T}$, was set to 0.03 rad/s. The controller was found to be run at 9.1 Hz. An explanatory video that demonstrates the test scenarios is given as a multimedia attachment in Supplementary Materials.

### 4.2. Results

**Figure 12:**
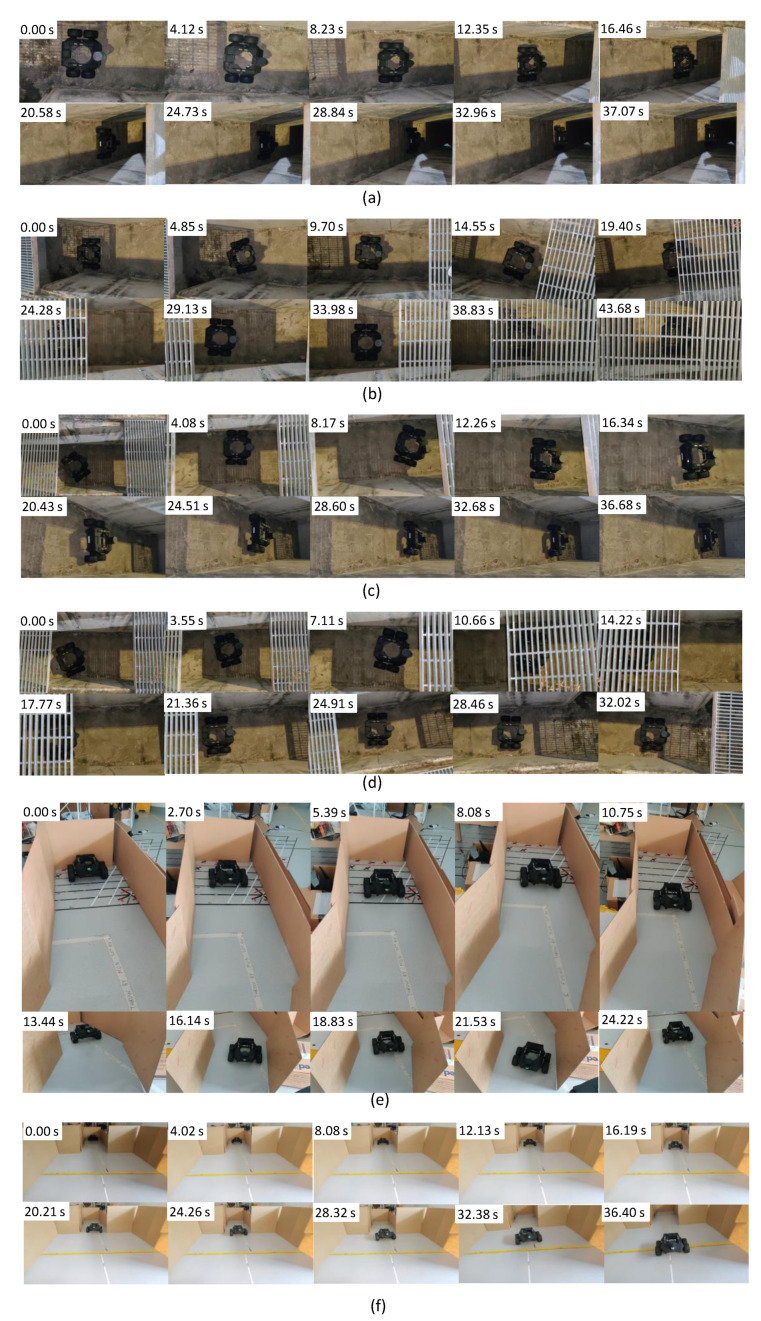
Arrangement of the test scenarios and the sequence of the robot’s movement. (**a**): Scenario ‘a’ where no initial heading and offset, (**b**): Scenario ‘b’ where there was an initial offset with no heading error, (**c**): Scenario ‘c’ where there was an initial error with no offset, (**d**): Scenario ‘d’ where the initial offset accompanied by a heading error, (**e**): Scenario ‘e,’ where a simulated situation of an angled drain, and (**f**): Scenario ‘f’ where the environment has a sudden change in width. It should be noted that Scenarios ‘a’ to ’d’ were conducted on a drain, and Scenarios ‘e’ and ‘f’ were conducted in a mock drain environment constructed in a laboratory setting.

**Figure 13:**
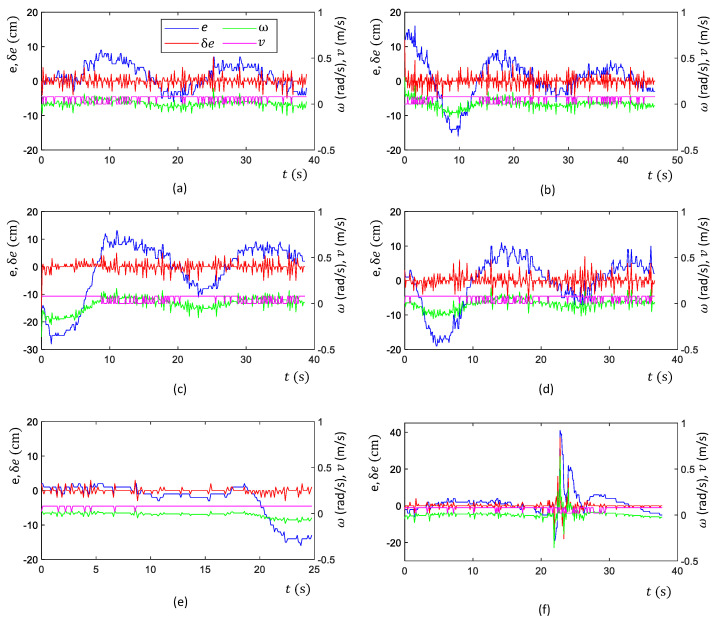
Variation of inputs and outputs of decision-making process of navigating in the middle of a drain in the considered test scenarios. (**a**) Scenario ‘a’, (**b**) Scenario ‘b’, (**c**) Scenario ‘c’, (**d**) Scenario ‘d’, (**e**) Scenario ‘e’, and (**f**) Scenario ‘f’.

**Figure 14:**
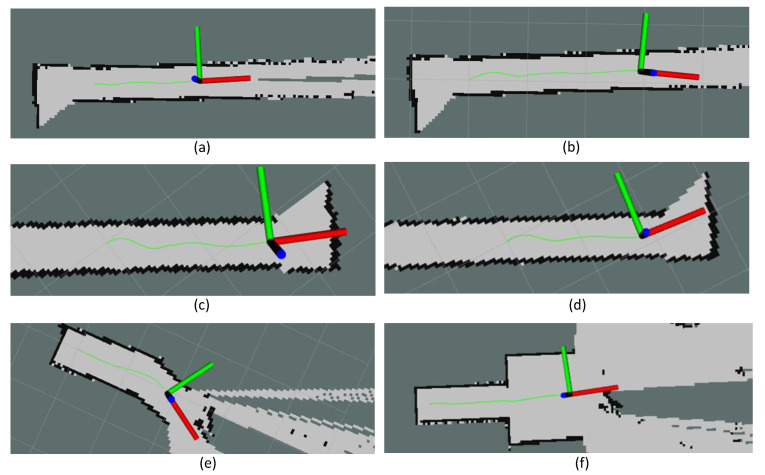
Traced path of the robot during the test scenarios. (**a**) Scenario ‘a’, (**b**) Scenario ‘b’, (**c**) Scenario ‘c’, (**d**) Scenario ‘d’, (**e**) Scenario ‘e’, and (**f**) Scenario ‘f’.

In test scenario ‘a’, the robot was placed in the drain with no heading error or offset from the middle, as shown in Figure 12a. Then, the navigation of the robot was initiated. The corresponding variations of the inputs and output of the controller in this scenario are given in Figure 13a. According to the plots, the error of the robot, *e*, and a few oscillations of *e* could be noted. However, the magnitude of the oscillations was trivial, leading to a small Root Mean Squared Error (RMSE) (RMSE = 3.8 cm). Thus, the performance of the controller was acceptable in this scenario. The traced path of the robot in this scenario is shown in Figure 14a. This path also confirms that the robot was properly moved in the middle of the drain.

Test scenario ‘b’ was designed to validate the ability of the controller to recover a possible offset of the robot from the middle during an inspection process. In this regard, the robot was initially placed in the drain with an offset with the middle. However, the initial heading of the robot was parallel to the drain. The sequence of snapshots of the robot taken during this case is shown in Figure 12b. The variation of *e* along with other inputs and outputs of the controller is given in Figure 13b. Initially, the robot had a higher *e*. With the actions of the controller, *e* was lowered quickly. After that, a few oscillations of *e* similar to test scenario ‘a’ can be seen. The traced path of the robot shown in Figure 14b also agrees with these observations. The robot RMSE in this case was also low at 5.9 cm, indicating the proposed controller’s ability to recover from a possible offset from the middle during operations.

There can be situations of heading errors with no offsets. Scenario ‘c’ was designed to evaluate the recovery ability of such situations. Thus, the robot was initially placed in the middle with a heading nonparallel to the drain, as shown in Figure 12c. A behaviour similar to scenario ‘b’ could be observed in variations of *e* (see Figure 13c), where the robot successfully recovered from the situation and moved along the middle of the drain (see the robot path given in Figure 14c). These results confirm the ability of the controller to recover from incorrect heading scenarios.

In scenario ‘d’, the robot was initially placed in the drain with both offset and heading error since the robot should have the ability to effectively cope with such situations (see Figure 12d). Similar to earlier cases, the robot successfully recovered from the initial errors and then moved along the middle of the drain, as shown in Figure 14d. The RMSE was 7.4 cm in here. These results confirm the proposed controller’s ability to maintain the robot in the middle of a drain even though it had to recover from an offset and heading error.

A drain segment with an angled direction was considered as scenario ‘e’ to evaluate the behaviour of the controller in such events. This scenario was conducted considering a simulated drain setting constructed in a laboratory setting, as shown in Figure 12e. According to the obtained results, the robot was capable of turning toward the new direction when it entered the angled segment (see Figure 14f). However, the robot had an offset and heading error when it entered into this segment. This segment is similar to scenario ‘d’ where the robot was successful with coping. Thus, it can be considered that the proposed control is capable of coping with angled drain directions.

Test scenario ‘f’ represented a situation where there is a sudden change in the width of a drain, as shown in Figure 12f. This scenario was also constructed within the laboratory setting. According to the plot shown in Figure 13f, a sudden change of *e* could be noted when the robot passed through the sudden width change. However, the robot could successfully manage the spikes of *e* without triggering undesired control actions and moved in the middle, as shown in Figure 14f.

In all test scenarios considered in the experiments, the proposed controller successfully navigated the robot in the middle of the drain. The considered test scenarios span most of the probable situations often encountered by a robot intended for drain inspection. Therefore, the proposed controller’s effectiveness in maintaining the robot in the middle of the drain when moving for the inspection can be concluded. Therefore, the proposed robot design and the controller would be helpful in improving the productivity of the robot-aided inspection process of drains, which is crucial for public health, safety, and for avoiding flash floods.

### 4.3. Discussion

Due to the rough nature of the drain walls, the range sensor information could include noises. The high-frequency variations of *e* observed in the plots are due to the noises. Especially, very large sudden variations of *e* could be observed in test scenario ‘f’ between 21–25 s even though the robot moved in the middle (robot’s movement in the middle without such variations can be confirmed from the map shown in Figure 14f). These variations were due to the sensor noises caused by uncertain characteristics of the side walls such as vertical slants, reflections, unevenness and impurities. Even though the proposed controller experienced imprecise input information from the sensors, the controller was capable of adequately navigating the robot in the middle of the drain. These observations confirm the sensor uncertainty handling ability of the proposed controller.

The fuzzy logic controller has no novelty from the perspective of fuzzy mathematics. However, this work uses a Mamdani-type fuzzy inference system as a framework for a novel application where a compact drain inspection robot is autonomously navigated along a drain while maintaining its position in the middle of the drain. The proposed controller determines the control actions of the robot, and the angular and linear velocity of the robot, based on the range sensor information, which consists of side clearances. Moreover, this work produces a novel control criterion for a drain inspection robot to improve its productivity. Very little work has been conducted on developing drain inspection robots, and a method with similar capabilities that can be used for performance comparison could not be found. Therefore, a performance comparison of the proposed robot and the existing work is not feasible. The movement of the robot along the drain with a sufficient side clearance to avoid collisions was considered the criterion that defined the controller’s success in a given scenario. In addition to that, the RMSE was used as a performance indicator where the average RMSE during the test cases was 6.6 cm. This amount of average deviation from the middle of the drain is acceptable in terms of the robot’s dimensions and the drain. Therefore, the proposed robot with the controller is helpful for complementing the drain inspections.

## 5. Conclusions

Robots have been explored for drain inspections to resolve the shortcomings of human labour-based approaches. A robot designed for drain inspections should cope with harsh environmental conditions such as rough terrain and confined space availability. Thus, the development of robots for drain inspection is challenging, and the existing robotics solutions for drain inspections require improvements.

This paper proposed a novel design of a drain inspection robot. The robot is equipped with a manually reconfigurable wheel axel mechanism that can be used to adapt the robot clearance height and length for the terrain conditions of a drain. A fuzzy controller is introduced to position the robot in the middle while moving along a drain to complement the inspection process.

Experiments have been conducted to validate the proposed robot design and the controller by deploying the robot to a drain setting. According to the experimental results, the proposed controller is effective in maintaining the robot in the middle while moving for inspections. Therefore, the findings of this work would be beneficial for improving drain inspection. It is expected to conduct experiments on drain settings with a stream of water in the future to evaluate the behaviour and determine the required adaptations. Furthermore, explorations on multirobot coordination for drain inspection are proposed for future work.

## Figures and Tables

**Table 1:** Comparison of the proposed design with the existing drain inspection robot.

Robot	Design Requirements *					
	a	b	c	d	e	f
PIRAT [16]	High	Low	Yes	High	Yes	Yes
KARO [17]	High	Low	Yes	High	Yes	Yes
KANTARO [18]	High	Low	Yes	High	Yes	Yes
KURT [19]	High	Low	Yes	High	Yes	Yes
MAKRO [20]	Medium	Low	No (lengthy)	Low	Yes	Yes
Tarantula [15]	Low	High	No (bulky)	Low	Yes	Yes
Raptor (proposed design)	High	High	Yes	High	Yes	Yes

* The labels are defined as follows; a: ability to manoeuvre in narrow drains, b: suitable to travel in various terrain, c: lightweight and compact, d: capability to make Sharp turns, e: flexibility to mount sensors and actuators, and f: capability to carry a payload.

**Table 2:** Rule base of the fuzzy logic system.

δe\e	L	LL	M	LR	R
NM	R	R	R	LR	M
NS	R	R	LR	M	LL
Z	R	LR	M	LL	L
PS	LR	M	LL	L	L
PM	M	LL	L	L	L

## Supplementary Materials

The following are available online at https://www.mdpi.com/article/10.3390/s21175742/s1.
